# *SLC7A11*-mediated cell death mechanism in cancer: a comparative study of disulfidptosis and ferroptosis

**DOI:** 10.3389/fcell.2025.1559423

**Published:** 2025-06-04

**Authors:** Wen-Wen Zhu, Ying Liu, Ze Yu, Hong-Qiang Wang

**Affiliations:** ^1^ Cancer Chemotherapy Center, Zhoushan Hospital, Wenzhou Medical University, Zhoushan, Zhejiang, China; ^2^ Department of pharmacy, Zhoushan Hospital, Wenzhou Medical University, Zhoushan, Zhejiang, China; ^3^ The laboratory of Cytobiology and Molecular Biology, Zhoushan Hospital, Wenzhou Medical University, Zhoushan, Zhejiang, China

**Keywords:** SLC7A11, disulfidptosis, ferroptosis, cancer, amino acid transport

## Abstract

SLC7A11: also known as Sodium-Coupled Neutral Amino Acid Transporter 11, is an essential transporter protein that plays a vital role in the intracellular transport of amino acids, especially cysteine. Recently, its involvement in cell death mechanisms has attracted considerable interest, underscoring its significance in both normal physiological functions and various disease states. This paper aims to provide a thorough overview of current understanding of the role of *SLC7A11* in two specific types of cell death: disulfidptosis and ferroptosis. We highlight the dual functions of *SLC7A11* in these processes, examining how its activity changes under different pathological conditions and what this means for potential therapeutic approaches. In this study, we clarify the regulatory mechanisms governing *SLC7A11* and its influence on cell death, ultimately presenting new insights that could guide future research in targeted therapies.

## 1 Introduction

The *SLC7A11* gene, commonly referred to as the cystine/glutamate antiporter, is essential for cellular amino acid metabolism, particularly in transporting cystine into cells, which is crucial for synthesizing glutathione (GSH). This antioxidant is important for maintaining the balance of redox within cells and protecting them from oxidative stress. *SLC7A11* is found in various tissues, with significant expression in the brain, liver, and kidney, where it helps regulate glutathione levels. The proper functioning of *SLC7A11* is vital for preventing oxidative damage, and when it is dysregulated, it has been linked to several diseases, including cancer and neurodegenerative disorders. In terms of cell metabolism, SLC7A11 not only aids in cystine uptake but also affects the synthesis of other amino acids, thus playing a crucial role in maintaining cellular homeostasis and helping cells adapt to stressful conditions (Koppula et al., 2018).

Cell death mechanisms can be categorized into several types, including apoptosis, necrosis, autophagic cell death, and regulated forms like ferroptosis and disulfidptosis. Each type of cell death has distinct biological functions and characteristics. For instance, apoptosis is a form of programmed cell death marked by cell shrinkage and chromatin condensation, while necrosis is characterized by cell swelling and eventual rupture. Ferroptosis, a newly recognized type of regulated cell death, involves the buildup of lipid peroxides due to iron-dependent processes, resulting in cellular damage and death (Dixon et al., 2012). In contrast, disulfidptosis is an emerging form of cell death that arises from the accumulation of intracellular disulfides, particularly under conditions of glucose deprivation (Liu et al., 2023). Understanding these mechanisms is essential for clarifying their roles in various physiological and pathological contexts, such as cancer, neurodegeneration, and other diseases.

Ferroptosis and disulfidptosis, with different processes, exhibit some similarities, especially in their dependence on oxidative stress and the involvement of specific metabolic pathways. Ferroptosis is mainly driven by lipid peroxidation and is closely associated with iron availability and the status of antioxidant defenses, particularly GSH system, which is significantly affected by the activity of *SLC7A11*. On the other hand, disulfidptosis is characterized by the accumulation of disulfides and is influenced by glucose metabolism.

In both ferroptosis and disulfidptosis, the regulation of redox status and the equilibrium between pro-oxidant and antioxidant mechanisms play a crucial role in determining cell fate. The biological importance of these cell death pathways is underscored by their potential implications for therapeutic strategies, especially in cancer treatment, where targeting these pathways could improve the effectiveness of therapies (Mao et al., 2024).

*SLC7A11* is becoming increasingly recognized as a crucial factor in both the ferroptosis and disulfidptosis, affecting how cells respond to oxidative stress and metabolic challenges. In the case of ferroptosis, *SLC7A11* plays a vital role in maintaining intracellular levels of cysteine, which are essential for the synthesis of GSH and for the cell’s antioxidant defenses. When *SLC7A11* is inhibited or its expression is reduced, cells are more vulnerable to ferroptosis because of decreased GSH levels and the buildup of lipid peroxides. On the other hand, in disulfidptosis, elevated levels of *SLC7A11* can result in increased disulfide stress, especially when intracellular glucose is scarce. This indicates *SLC7A11* may act as a regulatory center that integrates signals from both metabolic and oxidative stress pathways, potentially revealing new therapeutic targets for diseases associated with abnormal cell death (Koppula et al., 2021). Gaining a deeper understanding of SLC7A11’s dual role in these cell death processes could lead to innovative strategies for influencing cell fate in various diseases, particularly in the cancer treatment, where the ability to evade cell death is a key feature of tumor progression.

## 2 SLC7A11: Structure, function, and role in amino acid transport

### 2.1 Structure and function of SLC7A11

*SLC7A11*, commonly referred to as xCT, is part of the solute carrier family seven and is essential for transporting cystine and glutamate across cell membranes. This protein is structured with 12 transmembrane domains create a channel, allowing cystine to enter the cell while simultaneously exchanging it for glutamate. This transport process is crucial for maintaining the balance of redox within the cell. It plays a significant role in synthesizing glutathione, which is a vital antioxidant that helps protect cells from oxidative stress. Additionally, *SLC7A11* is important in preventing ferroptosis, a specific type of regulated cell death that relies on iron and is marked through the peroxidation of lipids (Lin et al., 2020; Jiang and Sun, 2024). The expression of *SLC7A11* is meticulously regulated by a range of oncogenes and tumor suppressors, highlighting its crucial role in tumor biology, especially regarding cancer progression and the development of resistance to treatments (Zhang et al., 2021; Zhang et al., 2019). Notably, the overexpression of *SLC7A11* has been associated with unfavorable outcomes in various cancers, suggesting that it may serve as a promising therapeutic target.

SLC7A11 primarily functions to transport cystine into cells while exporting glutamate, which is essential for maintaining a proper balance of these amino acids within the cell. This transport mechanism is especially vital in the tumor microenvironment, where cancer cells frequently face altered metabolic needs and heightened oxidative stress. The expression level of SLC7A11 is influenced by various factors, such as oxidative stress levels, nutrient availability, and signaling pathways that involve Nrf2. Additionally, the dysregulation of *SLC7A11* has been linked to several pathological conditions, including neurodegenerative diseases and cancer, underscoring its importance in cellular metabolism and survival (Wang et al., 2024a; Wang C. et al., 2023).

### 2.2 Role of *SLC7A11* in amino acid transport

*SLC7A11* is crucial for the transport of amino acids, especially in the exchange of cystine and glutamate. This transport process is vital for glutathione production, an important antioxidant that safeguards cells against oxidative damage and plays a key role in regulating ferroptosis (Niu et al., 2021). In cancer cells, the upregulation of SLC7A11 is often observed, which allows these cells to thrive in environments with high oxidative stress through maintaining adequate levels of cysteine for glutathione production (Lin et al., 2020). The ability of *SLC7A11* to modulate cellular redox status not only influences cell survival but also impacts the efficacy of various cancer therapies, as inhibiting *SLC7A11* can sensitize tumor cells to ferroptosis and enhance the effects of chemotherapy (Ouyang et al., 2022). The interaction between *SLC7A11* and various metabolic pathways highlights its critical role in cancer metabolism, positioning it as a promising target for therapeutic strategies designed to disrupt the metabolic adaptations that cancer cells undergo (Lee and Roh, 2022). Understanding the role of *SLC7A11* in amino acid transport offers valuable insights into its wider implications in cancer biology and potential therapeutic strategies.

## 3 Mechanism of disulfidptosis

### 3.1 Definition and biological Basis of disulfidptosis

**FIGURE 1:**
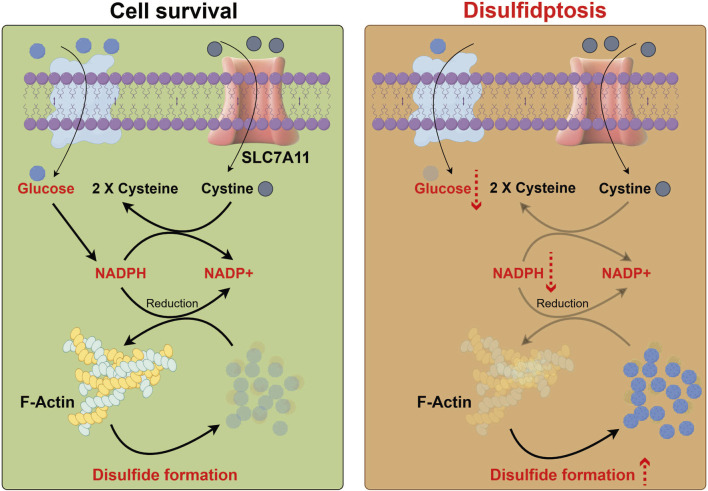
Process diagram of disulfidptosis. Glucose enters cells, while cysteine is taken up via SLC7A11 and reduced to two cysteine molecules. Glucose metabolism produces NADPH, crucial for reducing F-actin and maintaining cell stability. Lack of glucose depletes NADPH, preventing effective reduction of F-actin, leading to its oxidation, excessive disulfide bond formation, and cell death.

Disulfidptosis is a newly recognized type of regulated cell death that occurs due to the abnormal buildup of intracellular disulfides, which ultimately results in cellular dysfunction and death (Figure 1). This phenomenon is especially significant in various cancers, where disulfide stress can arise from metabolic changes, such as a lack of glucose. The underlying biological mechanisms of disulfidptosis involve a disruption in cellular redox balance, primarily influenced by SLC7A11. This protein is crucial for transporting cystine and synthesizing glutathione. When intracellular disulfide levels rise, they can cause oxidative stress and lipid peroxidation, leading to cell death. This process sets disulfidptosis apart from other forms of cell death, such as apoptosis. Recent research has underscored the need to comprehend the molecular mechanisms of disulfidptosis, particularly regarding cancer treatment, as targeting disulfide metabolism could open up new therapeutic strategies (Liu et al., 2023; Zhang et al., 2021).

### 3.2 Specific mechanism of SLC7A11 in disulfidptosis

The abnormal accumulation of intracellular disulfides in SLC7A11^high^ cells during glucose deprivation triggers a novel form of cell death, which is distinct from both the apoptosis and ferroptosis. This phenomenon has been designated as disulfidptosis by the research workers. Investigations utilizing chemical proteomics and cellular biology revealed that glucose starvation in SLC7A11^high^ cells leads to the formation of aberrant disulfide linkages in actin cytoskeletal proteins, resulting in F-actin disintegration in a manner dependent on SLC7A11. CRISPR screening and functional analyses indicated that the disruption of the WAVE regulatory complex, which facilitates actin polymerization and the formation of lamellipodia, inhibits disulfidptosis, whereas the continuous activation of Rac enhances it. Additional findings demonstrated that glucose transporter inhibitors provoke disulfidptosis in SLC7A11^high^ cancer cells and impede the growth of SLC7A11^high^ tumors (Liu et al., 2023; Shuai et al., 2020).

## 4 Ferroptosis mechanism

**FIGURE 2:**
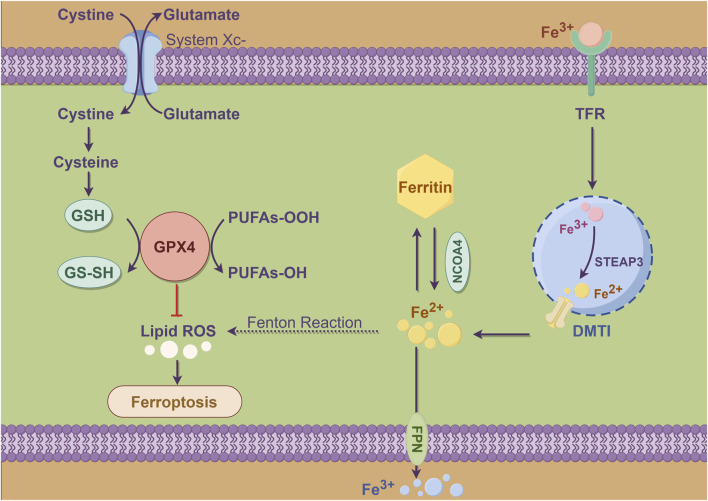
Schema diagram of metabolic pattern of ferroptosis in cells. Cysteine enters cells via system Xc-, exchanging with glutamate, and is reduced to GSH, which converts to GS-SH and is reduced by GPX4 to protect membranes from oxidative damage. Inhibition of GPX4 or depletion of GSH prevents effective reduction of lipid peroxides, leading to lipid peroxidation and increased ROS, causing ferroptosis. Iron ions enter through TFR, stored in ferritin or transported by DMT1, and can generate hydroxyl radicals via the Fenton reaction, promoting lipid peroxidation. Excess iron or imbalance in metabolism can worsen ferroptosis, with NCOA4 facilitating iron release from ferritin and STEAP3 influencing its reduction or oxidation.

Ferroptosis is a newly identified type of regulated cell death that is marked by the buildup of lipid peroxides and a reliance on iron. In contrast to apoptosis, which is mainly controlled by caspases, ferroptosis occurs due to oxidative stress and the breakdown of the cell’s antioxidant defenses (Figure 2). This process particularly involves the depletion of glutathione and activity of the enzyme glutathione peroxidase 4 (GPX4) (Hu et al., 2023). This unique form of cell death plays a significant role in various pathological conditions, including cancer, and stroke (Dong et al., 2024). The mechanism of ferroptosis encompasses several critical processes, including the accumulation of ROS, lipid peroxidation, and the disruption of iron homeostasis. When there is an imbalance between pro-oxidants and antioxidants, leading to oxidative stress, cells initiate ferroptosis. This process is typically characterized by distinct morphological changes, such as the shrinkage of mitochondria and an increase in membrane permeability (Wang et al., 2020). Researches have highlighted the involvement of various cellular pathways, such as autophagy and ubiquitin-proteasome system, in the regulation of ferroptosis. This indicates ferroptosis is influenced by a complex interplay of multiple cellular processes (Hu et al., 2023).

### 4.1 Role of SLC7A11 in ferroptosis and its regulatory mechanisms

SLC7A11 plays a crucial role in regulating ferroptosis, acting mainly as a cystine/glutamate antiporter that enables uptake of cystine into cells. Once inside, cystine is converted into cysteine, which is essential for the synthesis of glutathione. Elevated expression levels of *SLC7A11* are linked to resistance against ferroptosis, as this mechanism helps sustain intracellular glutathione levels, offering protection to cells from oxidative damage (Hu et al., 2023). In various cancers, *SLC7A11* is often overexpressed, contributing to tumor growth and survival by inhibiting ferroptosis (Liu et al., 2022). The regulation of SLC7A11 is intricate and encompasses various mechanisms, such as transcriptional regulation influenced by oncogenic pathways and post-translational modifications. A notable example is deubiquitylase OTUB1, which was recognized as a stabilizing factor for SLC7A11. By inhibiting its degradation, OTUB1 plays a key role in enhancing ferroptosis resistance in cancer cells (Liu et al., 2019). MicroRNAs and m6A RNA modifications have been found to play a significant role in regulating *SLC7A11* expression, introducing an additional layer of control over this important gene. The complex regulatory mechanisms associated with *SLC7A11* underscore its critical role not only in the process of ferroptosis but also within the larger framework of cancer biology and the challenges of therapeutic resistance.

## 5 SLC7A11 and tumor cell death

SLC7A11 has been linked to the ferroptosis and disulfidptosis. Ferroptosis is a form of cell death driven by iron-dependent lipid peroxidation, and it has emerged as a critical mechanism for tumor suppression. Elevated levels of SLC7A11 are frequently associated with resistance to ferroptosis, which allows cancer cells to survive despite oxidative stress that would typically induce cell death. For example, in cases of multiple myeloma, cells that express high levels of SLC7A11 showed increased sensitivity to ferroptosis induced by erastin, highlighting the complex role of SLC7A11 in influencing cell fate when faced with therapeutic challenges (Zhang et al., 2024). Moreover, the involvement of SLC7A11 in disulfidptosis, a recently identified form of cell death, underscores its dual role in either promoting cell survival or inducing cell death, which is contingent upon specific cellular context and the nature of external stressors (Liu et al., 2024). The complexity of this situation highlights the potential benefits of targeting SLC7A11 in cancer therapies. The advantages and prospective therapeutic benefits are manifested through:

1) Targeted modulation of oxidative stress mechanisms: through inhibiting SLC7A11, levels of reactive oxygen species (ROS) can be heightened, leading to the selective demise of tumor cells dependent on antioxidant mechanisms (Wang T. et al., 2023; Wang et al., 2024b);
2) Augmented effectiveness of combination therapies: For instance, the pairing of MLN4924 (a CRL3 inhibitor) with the ferroptosis inducer IKE can collaboratively suppress tumor proliferation by stabilizing SLC7A11 while concurrently obstructing its function (Zhou et al., 2024);
3) Reversal of the immunosuppressive tumor milieu: In lung adenocarcinoma, diminished expression of SLC7A11 correlates with heightened infiltration of CD8^+^ T cells, implying that its inhibition could bolster responses to immunotherapeutic approaches (Shan et al., 2023).

In conclusion, the intricacy of SLC7A11 in cell death is attributable to its regulation of oxidative stress, ferroptosis and multi-pathway interactions, which offers avenues for multifaceted intervention and numerous entry points. Targeting SLC7A11 has the potential to disrupt the distinctive metabolic weaknesses of cancer cells, enhance the sensitivity of conventional therapies, and ameliorate the immune microenvironment, rendering it a treatment strategy with expansive promise. Future investigations must further clarify its dynamic regulatory framework and refine combination therapy protocols for practical applicability in clinical settings.

### 5.1 Current status of drug development targeting SLC7A11

SLC7A11 is frequently found to be overexpressed in various types of cancer, which plays a significant role in promoting tumor growth. This overexpression aids in production of glutathione, an essential antioxidant that helps shield cells from oxidative stress and enhances their resistance to ferroptosis, a regulated form of cell death linked to iron-dependent lipid peroxidation (Li et al., 2022). Recent research has underscored the potential benefits of targeting SLC7A11 in cancer treatment. By inhibiting its function, it may be possible to make tumor cells more susceptible to ferroptosis and improve the effectiveness of chemotherapy (Ouyang et al., 2022).

Several pharmacological agents have been developed to inhibit SLC7A11, with erastin being a notable example. This compound directly targets the cystine/glutamate antiporter, resulting in reduced cystine uptake and the subsequent induction of ferroptosis in cancer cells. Furthermore, the discovery of small molecules that can selectively inhibit *SLC7A11* has paved the way for new therapeutic strategies. For example, L-selenocystine has demonstrated the ability to exploit the vulnerabilities associated with *SLC7A11* overexpression, leading to increased oxidative stress and cell death in cancer cells that depend on this transporter for their survival (Tan et al., 2023).

The role of microRNAs in regulating *SLC7A11* expression has become a promising avenue for drug development. For instance, miR-148a-3p has been recognized as a tumor suppressor that negatively influences *SLC7A11*, thereby promoting ferroptosis in colorectal cancer cells. This finding indicates restoring the expression of specific microRNA may represent a novel therapeutic strategy to improve effectiveness of current cancer treatments by specifically targeting SLC7A11.

### 5.2 Prospects of SLC7A11 in clinical treatment

Clinical implications of targeting SLC7A11 are substantial, particularly due to its connection with various malignancies and its involvement in mediating resistance to standard therapies. Elevated levels of SLC7A11 expression have been linked to unfavorable outcomes in multiple cancers, such as ovarian cancer. This correlation suggests that SLC7A11 could be an important biomarker for stratifying patients and guiding treatment choices (Fantone et al., 2024).

Emerging evidence indicates that inhibiting SLC7A11 can increase the sensitivity of cancer cells to ferroptosis and other cell death forms, which may help to overcome therapeutic resistance. For example, in ovarian cancer, targeting SLC7A11 has been demonstrated to induce ferroptosis, offering a potential strategy for treating patients who are resistant to standard chemotherapy. Additionally, combining SLC7A11 inhibitors with other therapeutic agents, such as chemotherapy or immunotherapy, could result in synergistic effects, thereby enhancing treatment outcomes for patients with advanced-stage cancers (Hong et al., 2021).

The advancement of personalized medicine strategies that take into account the expression levels of SLC7A11 and its related pathways has the potential to improve the efficacy of cancer treatments. For instance, the patients exhibiting elevated SLC7A11 expression may respond better to therapies designed to specifically inhibit this transporter, whereas individuals with lower levels might achieve satisfactory outcomes with standard treatment options, eliminating the necessity for SLC7A11 inhibition.

In summary, potential of SLC7A11 as a therapeutic target in clinical practice appears to be promising. Current research is dedicated to understanding its involvement in cancer progression and the mechanisms behind resistance, as well as exploring how targeted therapies could enhance patient outcomes. As we deepen our knowledge of SLC7A11, it is expected that new therapeutic strategies will develop, leading to more effective cancer treatments that are customized to meet the specific needs of individual patients.

### 5.3 Compare disultioptosis with ferroptosis

Based on the aforementioned studies, we meticulously examined the distinctions between disulfidptosis and ferroptosis from several perspectives, including preferences in tumor treatment, pivotal regulatory molecules, metabolic foundations, triggering conditions, physiological and pathological correlations, therapeutic implementations, and detection methodologies. The more detailed comparison and description of the reference Supplementary Material S1.

The primary aspects are as follows:

1) Metabolic essence: Ferroptosis is contingent upon iron and lipid peroxidation, whereas disulfide cell death relies on an imbalance in cystine metabolism and stress from disulfide bonds.
2) Therapeutic window: Ferroptosis is optimal for tumors characterized by elevated lipid metabolism, while disulfide death is more suitable for tumors exhibiting overexpression of SLC7A11 and a deficiency in glucose.
3) Biomarkers for detection: Ferroptosis can be identified through products of lipid peroxidation, while disulfide death is monitored via the states of disulfide and NADPH metabolism.
4) Clinical translation: Drugs inducing ferroptosis have progressed to clinical use, whereas disulfide death remains in experimental phases, necessitating the development of specific inducers.

## 6 Conclusion

The clinical significance of SLC7A11 as a therapeutic target is substantial due to its role in essential processes like response to oxidative stress and the regulation of cell death. Interventions that aim to modify SLC7A11 activity could lead to innovative strategies to improve treatment effectiveness and address resistance mechanisms in cancer therapies, as well as help in reducing neurodegeneration. However, it is important to maintain a balanced viewpoint; although targeting SLC7A11 offers promising opportunities for intervention, it is crucial to take into account the potential off-target effects and the necessity for specificity in the development of drugs.

SLC7A11, as a core component of the cystine/glutamate transporter, has dual importance in cancer therapy: on one hand, its high expression helps tumor cells resist ferroptosis by maintaining glutathione synthesis, inhibiting lipid peroxidation, thereby promoting chemotherapy resistance and tumor survival, particularly in solid tumors such as colorectal cancer and glioblastoma, which are significantly associated with poor prognosis; on the other hand, under specific metabolic stress, the abnormal activation of SLC7A11 can lead to the toxic accumulation of cystine and NADPH depletion, triggering disulfidptosis, providing a selective killing strategy for targeting tumors with high SLC7A11 expression. Additionally, SLC7A11 influences the immune response to therapy by regulating the redox balance and immune cell infiltration (such as inhibiting CD8^+^ T cell activity) in the tumor microenvironment, and its expression is subject to multi-level regulation by epigenetic modifications (such as m6A, ubiquitination) and transcription factors (NRF2, ATF4), offering diverse targets for the development of small molecule inhibitors, metabolic interventions (glucose deprivation), or combination therapies (such as with immune checkpoint inhibitors). Despite challenges such as normal tissue toxicity and tumor heterogeneity, precise regulation of SLC7A11 has become a key direction for overcoming treatment resistance, enhancing sensitivity to ferroptosis, and optimizing metabolic-immune combination therapies.

In conclusion, exploring the dual role of *SLC7A11* in disulfidptosis and ferroptosis has significantly enhanced our understanding of cellular death pathways. As discussed, SLC7A11 is essential for regulating intracellular cystine levels, which are vital for synthesizing glutathione and maintaining antioxidant defenses. Additionally, it plays a crucial role in modulating ferroptotic processes. This dual functionality emphasizes the complexity and interconnectedness of cell death mechanisms and suggests potential therapeutic opportunities for targeting SLC7A11 in various cancers.

## References

[B1] Dixon S. J. Lemberg K. M. Lamprecht M. R. Skouta R. Zaitsev E. M. Gleason C. E. (2012). Ferroptosis: an iron-dependent form of nonapoptotic cell death. Cell 149 (5), 1060–1072. 10.1016/j.cell.2012.03.042 22632970 PMC3367386

[B2] Dong H. Ma Y. P. Cui M. M. Qiu Z. H. He M. T. Zhang B. G. (2024). Recent advances in potential therapeutic targets of ferroptosis‑associated pathways for the treatment of stroke (Review). Mol. Med. Rep. 30 (1), 128. 10.3892/mmr.2024.13252 38785160 PMC11134507

[B3] Fantone S. Piani F. Olivieri F. Rippo M. R. Sirico A. Di Simone N. (2024). Role of slc7a11/xCT in ovarian cancer. Int. J. Mol. Sci. 25 (1), 587. 10.3390/ijms25010587 38203758 PMC10779187

[B4] Hong T. Lei G. Chen X. Li H. Zhang X. Wu N. (2021). PARP inhibition promotes ferroptosis via repressing SLC7A11 and synergizes with ferroptosis inducers in BRCA-proficient ovarian cancer. Redox Biol. 42, 101928. 10.1016/j.redox.2021.101928 33722571 PMC8113041

[B5] Hu S. Chu Y. Zhou X. Wang X. (2023). Recent advances of ferroptosis in tumor: from biological function to clinical application. Biomed. Pharmacother. 166, 115419. 10.1016/j.biopha.2023.115419 37666176

[B6] Jiang Y. Sun M. (2024). SLC7A11: the Achilles heel of tumor? Front. Immunol. 15, 1438807. 10.3389/fimmu.2024.1438807 39040097 PMC11260620

[B7] Koppula P. Zhang Y. Zhuang L. Gan B. (2018). Amino acid transporter SLC7A11/xCT at the crossroads of regulating redox homeostasis and nutrient dependency of cancer. Cancer Commun. (Lond). 38 (1), 12. 10.1186/s40880-018-0288-x 29764521 PMC5993148

[B8] Koppula P. Zhuang L. Gan B. (2021). Cystine transporter SLC7A11/xCT in cancer: ferroptosis, nutrient dependency, and cancer therapy. Protein Cell 12 (8), 599–620. 10.1007/s13238-020-00789-5 33000412 PMC8310547

[B9] Lee J. Roh J. L. (2022). SLC7A11 as a gateway of metabolic perturbation and ferroptosis vulnerability in cancer. Antioxidants (Basel) 11 (12), 2444. 10.3390/antiox11122444 36552652 PMC9774303

[B10] Li S. Lu Z. Sun R. Zhang M. (2022). The role of SLC7A11 in cancer: friend or foe? Cancers (Basel) 14 (13), 5902. 10.3390/cancers14235902 35804831 PMC9264807

[B11] Lin W. Wang C. Liu G. Bi C. Wang X. Zhou Q. (2020). SLC7A11/xCT in cancer: biological functions and therapeutic implications. Am. J. Cancer Res. 10 (10), 3106–3126. 33163260 PMC7642655

[B12] Liu L. He J. Sun G. Huang N. Bian Z. Xu C. (2022). The N6-methyladenosine modification enhances ferroptosis resistance through inhibiting SLC7A11 mRNA deadenylation in hepatoblastoma. Clin. Transl. Med. 12 (5), e778. 10.1002/ctm2.778 35522946 PMC9076012

[B13] Liu T. Jiang L. Tavana O. Gu W. (2019). The deubiquitylase OTUB1 mediates ferroptosis via stabilization of SLC7A11. Cancer Res. 79 (8), 1913–1924. 10.1158/0008-5472.CAN-18-3037 30709928 PMC6467774

[B14] Liu X. Nie L. Zhang Y. Yan Y. Wang C. Colic M. (2023). Actin cytoskeleton vulnerability to disulfide stress mediates disulfidptosis. Nat. Cell Biol. 25 (3), 404–414. 10.1038/s41556-023-01091-2 36747082 PMC10027392

[B15] Liu X. Zhuang L. Gan B. (2024). Disulfidptosis: disulfide stress-induced cell death. Trends Cell Biol. 34 (4), 327–337. 10.1016/j.tcb.2023.07.009 37574347

[B16] Mao C. Wang M. Zhuang L. Gan B. (2024). Metabolic cell death in cancer: ferroptosis, cuproptosis, disulfidptosis, and beyond. Protein Cell 15 (9), 642–660. 10.1093/procel/pwae003 38428031 PMC11365558

[B17] Niu B. Liao K. Zhou Y. Wen T. Quan G. Pan X. (2021). Application of glutathione depletion in cancer therapy: enhanced ROS-based therapy, ferroptosis, and chemotherapy. Biomaterials 277, 121110. 10.1016/j.biomaterials.2021.121110 34482088

[B18] Ouyang S. Li H. Lou L. Huang Q. Zhang Z. Mo J. (2022). Inhibition of STAT3-ferroptosis negative regulatory axis suppresses tumor growth and alleviates chemoresistance in gastric cancer. Redox Biol. 52, 102317. 10.1016/j.redox.2022.102317 35483272 PMC9108091

[B19] Shan Q. Zhang C. Li Y. Li Q. Zhang Y. Li X. (2023). SLC7A11, a potential immunotherapeutic target in lung adenocarcinoma. Sci. Rep. 13 (1), 18302. 10.1038/s41598-023-45284-z 37880315 PMC10600206

[B20] Shuai Y. Ma Z. Yuan P. (2020)2024). Disulfidptosis: disulfide stress-induced novel cell death pathway. MedComm 5 (7), e579. 10.1002/mco2.579 PMC11214019 38948113

[B21] Tan S. L. W. Tan H. M. Israeli E. Fatihah I. Ramachandran V. Ali S. B. (2023). Up-regulation of SLC7A11/xCT creates a vulnerability to selenocystine-induced cytotoxicity. Biochem. J. 480 (24), 2045–2058. 10.1042/BCJ20230317 38078799

[B22] Wang C. Liu H. Xu S. Deng Y. Xu B. Yang T. (2023a). Ferroptosis and neurodegenerative diseases: insights into the regulatory roles of SLC7A11. Cell Mol. Neurobiol. 43 (6), 2627–2642. 10.1007/s10571-023-01343-7 36988772 PMC11410137

[B23] Wang H. Liu C. Zhao Y. Gao G. (2020). Mitochondria regulation in ferroptosis. Eur. J. Cell Biol. 99 (1), 151058. 10.1016/j.ejcb.2019.151058 31810634

[B24] Wang T. Liang S. Li Y. Wang X. Wang H. Guo J. (2023b). Downregulation of lncRNA SLC7A11-AS1 decreased the NRF2/SLC7A11 expression and inhibited the progression of colorectal cancer cells. PeerJ 11, e15216. 10.7717/peerj.15216 37077308 PMC10108855

[B25] Wang Z. Wang Y. Shen N. Liu Y. Xu X. Zhu R. (2024b). AMPKα1-mediated ZDHHC8 phosphorylation promotes the palmitoylation of SLC7A11 to facilitate ferroptosis resistance in glioblastoma. Cancer Lett. 584, 216619. 10.1016/j.canlet.2024.216619 38211651

[B26] Wang Z. Zong H. Liu W. Lin W. Sun A. Ding Z. (2024a). Augmented ERO1α upon mTORC1 activation induces ferroptosis resistance and tumor progression via upregulation of SLC7A11. J. Exp. Clin. Cancer Res. 43 (1), 112. 10.1186/s13046-024-03039-2 38610018 PMC11015652

[B27] Zhang W. Li Q. Zhang Y. Wang Z. Yuan S. Zhang X. (2024). Multiple myeloma with high expression of SLC7A11 is sensitive to erastin-induced ferroptosis. Apoptosis 29 (3-4), 412–423. 10.1007/s10495-023-01909-2 38001343

[B28] Zhang W. Sun Y. Bai L. Zhi L. Yang Y. Zhao Q. (2021). RBMS1 regulates lung cancer ferroptosis through translational control of SLC7A11. J. Clin. Invest 131 (22), e152067. 10.1172/JCI152067 34609966 PMC8592553

[B29] Zhang Y. Koppula P. Gan B. (2019). Regulation of H2A ubiquitination and SLC7A11 expression by BAP1 and PRC1. Cell Cycle 18 (8), 773–783. 10.1080/15384101.2019.1597506 30907299 PMC6527290

[B30] Zhou Q. Yu H. Chen Y. Ren J. Lu Y. Sun Y. (2024). The CRL3(KCTD10) ubiquitin ligase-USP18 axis coordinately regulates cystine uptake and ferroptosis by modulating SLC7A11. Proc. Natl. Acad. Sci. U. S. A. 121 (28), e2320655121. 10.1073/pnas.2320655121 38959043 PMC11252818

