# Supplementary material for: SLC7A11-mediated cell death mechanism in cancer: a comparative study of disulfidptosis and ferroptosis
Source: Front Cell Dev Biol. 2025 Jun 4;13:1559423. doi: 10.3389/fcell.2025.1559423 (PMC12174375; doi:10.3389/fcell.2025.1559423)
Supplement: Supplementary file 1 [file Table1.docx]

Supplementary table 1. Therapeutic Preferences for Different Tumors

| Aspect | Ferroptosis | Disulfidptosis |
| --- | --- | --- |
| Target Tumor Types | Liver cancer, renal cell carcinoma, triple-negative breast cancer (TNBC) | Glioblastoma, lung cancer, ovarian cancer (tumors with high SLC7A11 expression) |
| Therapeutic Advantage | Effective against therapy-resistant tumors with high lipid peroxidation | Selective killing of SLC7A11-high tumors under glucose-deprived microenvironments |

Supplementary table 2. Key Regulatory Molecules

| Aspect | Ferroptosis | Disulfidptosis |
| --- | --- | --- |
| Core Regulators | GPX4, SLC7A11, ACSL4, Nrf2 | SLC7A11, NADPH-generating enzymes (G6PD, IDH1), TXNRD1 |
| Suppressors | FSP1 (CoQ10-dependent), Ferritin (iron storage) | GLUT1 (glucose transport), ZDHHC8 (SLC7A11 palmitoylation) |

Supplementary table 3. Metabolic Basis

| Aspect | Ferroptosis | Disulfidptosis |
| --- | --- | --- |
| Core Pathways | Iron-dependent lipid peroxidation, GSH depletion | Cystine import via SLC7A11, NADPH insufficiency, disulfide stress |
| Key Metabolites | Lipid ROS (e.g., MDA, 4-HNE), Fe²⁺ | Accumulated cystine, NADPH/NADP+ imbalance |

Supplementary table 4. Triggering Conditions

| Aspect | Ferroptosis | Disulfidptosis |
| --- | --- | --- |
| Inducers | Erastin, RSL3, cystine deprivation | Glucose deprivation, SLC7A11 overexpression + cystine supplementation |
| Inhibitors | Iron chelators (e.g., deferoxamine), lipophilic antioxidants (vitamin E) | Glucose supplementation, NAC (cysteine donor) |

Supplementary table 5. Physiological & Pathological Roles

| Aspect | Ferroptosis | Disulfidptosis |
| --- | --- | --- |
| Physiological Role | Clearance of damaged cells, embryonic development | Redox homeostasis via cystine/cysteine cycling |
| Pathological Role | Neurodegeneration (Alzheimer’s, Parkinson’s), ischemia-reperfusion injury | Cancer metastasis, chemotherapy resistance |

Supplementary table 6. Therapeutic Applications

| Aspect | Ferroptosis | Disulfidptosis |
| --- | --- | --- |
| Clinical Strategies | Ferroptosis inducers (sorafenib, IKE), combination with immunotherapy | SLC7A11 inhibitors, glucose restriction + cystine loading |
| Challenges | Toxicity to normal tissues (liver, kidney), tumor heterogeneity | Dependency on glucose-deprived niches, precise targeting of SLC7A11-high tumors |

Supplementary table 7. Detection Methods

| Aspect | Ferroptosis | Disulfidptosis |
| --- | --- | --- |
| Biomarkers | Lipid peroxidation (C11-BODIPY, MDA assay), iron staining (Perls’ Prussian blue) | Disulfide accumulation (DTNB assay), NADPH/NADP+ ratio (fluorescence probes) |
| Functional Validation | GPX4 knockout, iron chelator rescue | SLC7A11 knockdown, glucose rescue experiments |

Supplementary table 8. Summary Table

| Aspect | Ferroptosis | Disulfidptosis |
| --- | --- | --- |
| Metabolic Basis | Iron-dependent lipid peroxidation | Cystine overload and disulfide stress |
| Key Regulators | GPX4, SLC7A11, ACSL4 | SLC7A11, NADPH enzymes, TXNRD1 |
| Inducing Conditions | GPX4 inhibition, cystine deprivation | Glucose deprivation + SLC7A11 overexpression |
| Therapeutic Targets | Liver cancer, TNBC | Glioblastoma, SLC7A11-high lung/ovarian cancers |
| Detection | Lipid ROS, iron accumulation | Disulfide levels, NADPH/NADP+ imbalance |
| Clinical Challenges | Organ toxicity, tumor resistance | Metabolic dependency, precise targeting |
